# Cardiac Structure and Function in Young Adults With Various Cardiometabolic Profiles

**DOI:** 10.7759/cureus.40524

**Published:** 2023-06-16

**Authors:** Swapnil D Parve, Albina V Sineglazova

**Affiliations:** 1 Primary Care and General Practice, Kazan State Medical University, Kazan, RUS; 2 Research and Development, Datta Meghe Institute of Higher Education & Research (Deemed to be University), Wardha, IND

**Keywords:** heart failure with preserved ejection fraction, adiposopathy, cardiometabolic diseases, visceral fat, obesity, echocardiographic parameters, cardiometabolic disease staging, cardiometabolic risk

## Abstract

Background

Cardiovascular diseases are a leading cause of mortality worldwide. Cardiometabolic abnormalities result in alterations in the myocardial structure and function. Limited data are available on these changes in young adults with various cardiometabolic risk profiles. The goal was to study the relationship between cardiometabolic risk and echocardiographic changes in young patients of both sexes in a Russian population, using a risk-based cardiometabolic disease staging (CMDS) system.

Methods

A total of 191 patients were included. The patients were classified into five groups based on the CMDS system. We gathered patient history and performed a physical exam, biochemical blood analysis, and echocardiography. Statistical analyses were performed using IBM SPSS Statistics for Windows, Version 23 (Released 2015; IBM Corp., Armonk, New York, United States).

Results

The median age of the participants was 35 (30.0-39.0) years. Elevated systolic and diastolic blood pressure and hypertriglyceridemia were more frequent (*p *< 0.05) in males than in females. An increase in the end-diastolic volume (EDV) and end-systolic volume (ESV) and a decrease in the ejection fraction were noted from CMDS 0 to 3. The EDV and ESV were associated with most cardiometabolic risk factors and strongly correlated with the visceral fat level, waist circumference, and body mass index. We identified a new subgroup as CMDS 3-overly high in patients with CMDS 3 and an excess level of visceral fat.

Conclusion

When designing strategies for cardiovascular disease prevention in young adults apart from CMDS parameters, bioimpedance analysis should be considered to assess the level of visceral fat, especially in individuals with CMDS 3 because they are at a higher risk of cardiac chamber enlargements. These results can be used to identify new dominants or phenotypes of heart failure with preserved ejection fraction.

## Introduction

Cardiovascular diseases remain a leading cause of mortality worldwide, including in Russia, despite recent advances in diagnostic and treatment approaches [1,2]. Even after achieving the target low-density lipoprotein cholesterol (LDL-c) levels, patients with coronary artery disease still have a residual risk of cardiovascular complications and death. This may be attributed to the presence of several cardiometabolic risk factors such as high blood pressure (BP), obesity, disorders of carbohydrate and lipid metabolism, and associated neurohumoral, dysmetabolic, and pro-inflammatory changes [3,4]. Owing to the nature of the association between these risk factors, evolving knowledge about cardiometabolic processes and advances in early diagnostic techniques warrant their constant monitoring for targeted preventive programs [3,5-7].

Cardiometabolic risk factors trigger alterations in cardiomyocyte metabolism, endothelial dysfunction, and microcirculation, resulting in changes in the myocardial structure and function [8-13]. The cardiometabolic-based chronic disease model proposed by Mechanick et al. considers adiposity and dysglycemia as key metabolic drivers, resulting in three major cardiovascular endpoints: coronary artery disease, atrial fibrillation, and chronic heart failure, especially heart failure with preserved ejection fraction (HFpEF) [14,15]. HFpEF is a heterogeneous syndrome, and due to its myriad presentation and lack of specific major society guidelines, its diagnosis is challenging. Several authors have suggested methods such as phenotyping of HFpEF subtypes; however, there is no consensus on any specific approach [16-18].

Since left ventricular dysfunction starts much earlier before overt heart failure, it is imperative to study early cardiac changes in patients with cardiometabolic risk factors [14,15,19]. There are limited data available on intracardiac hemodynamic changes in young adults, and they are primarily devoted to the study of structural and functional changes in the heart with only certain cardiometabolic risk factors [8,9,11-13,20]. Therefore, studying the characteristics of cardiometabolic profiles in a cohort of young adults using a validated instrument in conjunction with the study of intracardiac hemodynamics is of great interest.

The goal was to study the relationship between cardiometabolic risk and echocardiographic changes in young patients of both sexes in a Russian population, using a risk-based cardiometabolic disease staging (CMDS) system. 

## Materials and methods

Study setting and participants

This cross-sectional study was conducted at the Consultative Diagnostic Center of Aviastroytelniy District, a clinical center of the Department of Primary Care and General Practice of Kazan State Medical University in Kazan, Russia. The study was conducted between 2021 and 2022. In total, 191 patients were included in this study. 

Inclusion criteria

Inclusion criteria were individuals aged 25-44 years who provided voluntary informed consent to participate in this study.

Exclusion criteria

Exclusion criteria included refusal of the subject to participate in the study; patients with mental illness hampering the interview; the presence of verified cardiometabolic diseases (type 2 diabetes mellitus, coronary artery disease, congestive heart failure, atrial fibrillation, chronic kidney disease); antiphospholipid syndrome and autoimmune inflammatory diseases; the presence of verified oncology; decompensatory states of concomitant diseases or conditions (liver disease, kidney disease, etc.), acute infectious diseases, diseases of the endocrine system, and other diseases and conditions that are secondary causes of obesity; medical implants including a pacemaker, silicone implants, and metal prostheses; and pregnant and lactating women. 

Clinical and biochemical data collection

A detailed patient interview was conducted, with a thorough history and physical examination. The patient card included questions on demographic characteristics, medical history, family history, and psychosocial history, including questions on current smoking and physical activity. During physical examination, arterial BP was calculated as the mean of the second and third measurement out of three consecutive measurements. Height was measured on a certified stadiometer. Weight and body composition were evaluated using a Tanita BC-601 body composition monitor (‎Tanita Corporation, Japan). A visceral fat rating in the range of 1-12 was considered normal, whereas 13-59 was established as excess visceral fat [21]. Body mass index (BMI) was calculated as weight in kilograms divided by height in meters squared. Waist circumference was measured at the midpoint between the iliac crest and the lower edge of the ribcage [22].

The workup was performed using fasting venous blood samples in a single certified laboratory. The lipid profile (total cholesterol, triglycerides, high-density lipoprotein cholesterol (HDL-c), low-density lipoprotein cholesterol (LDL-c), non-HDL-c) and carbohydrate metabolism (fasting plasma glucose, oral glucose tolerance test, glycated hemoglobin, insulin) were checked using automated enzymatic methods on a Beckmann Coulter automated analyzer AU480 (Beckman Coulter Inc., Brea, USA) and Beckmann Coulter assays. In addition, we assessed insulin resistance according to the Homeostasis Model Assessment of Insulin Resistance to be >2.52. Serum high-sensitivity CRP (hsCRP) was measured using a high-sensitivity immunoturbidimetric method (CRP [Latex] Beckman Coulter, Japan), and levels >3 mg/L were considered elevated. The visceral adiposity index (VAI) was estimated with respect to age [23]. 

Echocardiographic assessment

A team of two trained and certified cardiologists together performed Doppler echocardiography on a Mindray DC-8 (Mindray Medical International Limited, Shenzen, China) machine in M- and B-modes with tissue Doppler imaging, as required, according to the guidelines [24]. We assessed left atrial volume and size, end-diastolic volume (EDV), end-systolic volume (ESV), ejection fraction (EF), stroke volume (SV), and cardiac output (CO). Based on structural measurements, we classified the types of ventricular remodeling [12]. The body surface area was calculated using the Du Bois formula to further estimate the LV mass index [25].

Evaluation of cardiometabolic risk

Cardiometabolic risk was evaluated based on the presence of risk factors, and patients were categorized according to the CMDS system [26,27]. Stage 0 individuals were metabolically healthy without any presence of risk factors; stage 1 (low risk) included individuals who had one or two of the following risk factors (RF1-RF4): (RF1) abdominal obesity established by waist circumference ≥80 cm and/or waist to hip ratio >0.85 in females and ≥94 cm and/or a waist to hip ratio >0.9 in males for Russian population [28]; (RF2) increased BP defined as systolic blood pressure ≥130 mmHg and/or diastolic blood pressure ≥85 mmHg or on antihypertensive therapy; (RF3) low HDL-c defined as HDL-c <1.3 mmol/L in females and <1.0 mmol/L in males or on lipid-lowering therapy; (RF4) fasting hypertriglyceridemia defined as triglycerides ≥1.7 mmol/l or on medication; stage 2 (medium risk) included subjects with one of the following: A1 - three or more metabolic risk factors listed in stage 1 (RF1-RF4), or with the presence of prediabetes, defined as (A2) impaired fasting glucose (IFG - venous glucose 6.1-6.9 mmol/L) or (A3) impaired glucose tolerance (IGT - 2-h venous glucose 7.8-11.0 mmol/L) i.e. metabolic abnormalities or prediabetes; stage 3 (high risk) included participants with two or three of the conditions listed in stage 2 (A1-A3) i.e. metabolic abnormalities + prediabetes; stage 4 (end-stage disease) when subject had diagnosed type 2 diabetes (T2D) and/or vascular disease (coronary artery disease, stroke, peripheral artery disease etc.). Individuals with stage 4 disease were excluded from this study.

Ethical approval

The study protocol conformed to the ethical guidelines of the 1975 Declaration of Helsinki and was approved by the Local Ethics Committee of Kazan State Medical University (protocol No. 6, dated June 22, 2021). All participants were informed about the study in a simple, easy-to-understand language and were assured of confidentiality. 

Statistical analysis

Statistical analyses were performed using IBM SPSS Statistics for Windows, Version 23 (Released 2015; IBM Corp., Armonk, New York, United States). The normality of continuous variables was assessed using the Kolmogorov-Smirnov test. Since the data were not normally distributed, non-parametric tests were used. The Mann-Whitney U-test was used to compare two independent groups, and the Kruskal-Wallis test was used to compare three or more groups. Continuous variables are presented as medians and interquartile ranges (IQR, 25th-75th percentile). Descriptive statistics were used to obtain frequencies and percentages for categorical variables. Statistical differences in categorical variables were tested using Pearson's Chi-square test or Fisher's exact test. Spearman’s (rs) correlation was used to study the correlation between cardiometabolic risk factors and echocardiographic parameters. The area under the curve (AUC) of receiver operator characteristic (ROC) curves were used. The AUC was calculated to assess the changes in hemodynamic parameters. The sensitivity and specificity values were calculated. The highest value of the sum of sensitivity and specificity, in favor of sensitivity, was used to determine the cut-off values for detecting changes in hemodynamic parameters. Differences were considered statistically significant set at p < 0.05 (two-tailed).

## Results

A total of 97 females (50.8%) and 94 males (49.2%) were included in this study. The median patient age was 35 (30.0-39.0) years. Elevated systolic blood pressure (SBP), diastolic blood pressure (DBP), and hypertriglyceridemia were more frequent (p < 0.05) in males than in females (p < 0.05; Table 1).

**Table 1:** Characteristics of study participants Note: n: number of participants with deranged parameters; %: proportion of subjects with deranged parameters presented as percent; p_1,2_:_ _difference in proportions between female (1) and male (2) based on the Chi-square test. Abbreviations: BMI: body mass index; SBP: systolic blood pressure; DBP: diastolic blood pressure; HDL-c: high-density lipoprotein cholesterol; IFG: impaired fasting glucose; IGT: impaired glucose tolerance.

Risk factors	Total (n=191)	Females (n = 97)	Males (n = 94)	p_1,2_
		1	2	
	n (%)	n (%)	n (%)	
BMI ≥ 25 Kg/m^2^	127 (66.5)	65 (67.0)	62 (66.0)	0.878
BMI ≥ 30 Kg/m^2^	64 (33.5)	34 (35.0)	30 (31.9)	0.646
Obesity Class I	39 (20.4)	19 (19.6)	20 (21.3)	0.923
Obesity Class II	17 (8.9)	10 (10.3)	7 (7.4)	0.475
Obesity Class III	8 (4.1)	5 (5.1)	3 (3.1)	0.479
Abdominal obesity	108 (56.5)	59 (60.8)	49 (52.1)	0.225
SBP ≥ 130 mm Hg	53 (27.7)	14 (14.4)	39 (41.5)	0.000
DBP ≥ 85 mm Hg	48 (25.1)	14 (14.4)	34 (36.2)	0.001
Low HDL-c (in Males < 1.0 mmol/L; Females < 1.2 mmol/L)	49 (25.7)	23 (23.7)	26 (27.7)	0.532
Hypertriglyceridemia (≥ 1,7 mmol/L)	36 (18.9)	11 (11.3)	25 (26.6)	0.007
IFG	4 (2.1)	1 (1.0)	3 (3.2)	0.297
IGT	64 (33.5)	38 (39.1)	26 (27.7)	0.092
Newly diagnosed type 2 diabetes mellitus	6 (3.1)	2 (2.6)	4 (4.3)	0.385

The distribution of participants according to CMDS is presented in Table 2. No statistically significant differences were observed in the age of the study subjects across different CMDS, both in the general and sex-based cohort (p = 0.096-0.568). Given the normal distribution of females and males, further analysis across various CMDS was conducted in the overall cohort. 

**Table 2:** Frequency of cardiometabolic risk according to the CMDS system Note: n: number of participants in a particular stage; %: proportion of subjects in a particular stage, presented as percentage; p_1,2_ the difference between female (1) and male (2) sex based on the Chi-square test. Abbreviations: CMDS: cardiometabolic disease staging.

CMDS	Females (n = 97)	Males (n = 94)	p_1,2_
	1	2	
	n (%)	n (%)	
CMDS 0	21 (21.6)	16 (17.0)	0.418
CMDS 1	35 (36.0)	34 (36.1)	0.553
CMDS 2	30 (30.9)	28 (29.8)	0.631
CMDS 3	9 (9.3)	12 (12.8)	0.309
CMDS 4	2 (2.0)	4 (4.3)	0.286

After workup, patients with type 2 diabetes mellitus and echocardiography confirmed heart failure with reduced ejection were excluded from the study and final analysis included 181 individuals. A detailed breakdown of cardiometabolic risk factors in various CMDS is presented in Table 3.

**Table 3:** Characteristics of cardiometabolic risk factors in various CMDS Note: n: number of participants with deranged parameters; %: proportion of subjects with deranged parameters presented as percent; p_1,2_:_ _significance between CMDS 0 and CMDS 1; p_1,3_ : significance between CMDS 0 and CMDS 2; p_1,4_ : significance between CMDS 0 and CMDS 3; p_2,3_ : significance between CMDS 1 and CMDS 2; p_2,4_ : significance between CMDS 1 and CMDS 3; p_3,4_ : significance between CMDS 2 and CMDS 3, calculated using the Mann‐Whitney test. NA: not available. Abbreviations: BMI: body mass index; SBP: systolic blood pressure; DBP: diastolic blood pressure; HOMA-IR: homeostatic model assessment of insulin resistance; IGT: impaired glucose tolerance; HDL-c - high-density lipoprotein cholesterol; non-HDL-c: non-high-density lipoprotein cholesterol; VAI: visceral adiposity index; CRP: C-reactive protein.

Cardiometabolic risk factor	CMDS 0 (n = 36)	CMDS 1 (n = 69)	CMDS 2 (n = 57)	CMDS 3 (n = 19)	p_1,2_	p_1,3_	p_1,4_	p_2,3_	p_2,4_	p_3,4_
	1	2	3	4						
	n (%)	n (%)	n (%)	n (%)						
SBP ≥130 mmHg	0	19 (27.5)	19 (32.8)	11 (52.4)	0.000	0.000	0.000	0.522	0.034	0.112
DBP ≥85 mmHg	0	17 (24.6)	17 (29.3)	13 (61.9)	0.001	0.000	0.000	0.554	0.002	0.008
HOMA-IR >2.52	1 (2.7)	15 (21.7)	14 (24.1)	14 (66.7)	0.009	0.005	0.000	0.748	0.000	0.000
IGT	0	0	19 (32.8)	16 (76.1)	NA	0.000	0.000	0.000	0.000	0.001
Excess level of visceral fat	0	4 (5.8)	2 (3.4)	5 (23.8)	0.135	0.249	0.002	0.548	0.016	0.005
Leptin >11.1 ng/mL	17 (46)	34 (49.2)	36 (6.2)	19 (90.5)	0.345	0.053	0.000	0.248	0.001	0.006
Total cholesterol ≥5 mmol/L	9 (24.3)	29 (42)	29 (50)	11 (52.3)	0.070	0.013	0.031	0.369	0.403	0.852
Triglyceride ≥1.7 mmol/L	0	4 (5.8)	14 (24.1)	15 (71.4)	0.135	0.001	0.000	0.003	0.000	0.000
Low HDL-c (in Males < 1.0 mmol/L; Females < 1.2 mmol/L)	0	16 (23.1)	18 (31)	14 (66.7)	0.001	0.000	0.000	0.291	0.000	0.005
LDL-c >3 mmol/L	18 (48.7)	39 (56.6)	34 (58.6)	18 (85.7)	0.438	0.341	0.005	0.812	0.015	0.025
Non-HDL-c >3.4 mmol/L	12 (32.4)	36 (52.1)	35 (60.3)	16 (76.1)	0.044	0.008	0.001	0.404	0.059	0.193
Atherogenicity index >3	4 (10.9)	24 (34.8)	27 (46.6)	18 (85.7)	0.007	0.000	0.000	0.171	0.000	0.002
VAI	0	3 (4,3)	14 (24.1)	17 (81)	0.198	0.001	0.000	0.001	0.000	0.000
CRP >3 mg/L	1 (2.7)	23 (33.3)	12 (20.7)	12 (57.1)	0.000	0.013	0.000	0.112	0.050	0.002

The cardiac structural and functional parameters in the general cohort were within reference values (Table 4). Nonetheless, an increase in EDV and ESV and a decrease in EF were noted from CMDS 0 to 3. This trend was further confirmed using a statistically significant Kruskal-Wallis test. The stroke volume was also higher in the groups with CMDS 2 and 3 than in those with CMDS 0. Similarly, a higher end diastolic dimension (EDD) was also revealed in patients from groups with higher cardiometabolic risk than in those with CMDS 0. A similar analysis was conducted for males and females, and the results were comparable to those of the general cohort.

**Table 4:** Characteristics of key structural and functional parameters of the heart in various CMDS Note: n: number of participants in a particular group; Me: median [interquartile range, 25th–75th percentile], p_K-W: _ p-value from the Kruskal–Wallis test. Abbreviations: LA: left atrium; LV: left ventricle; EDD: end-diastolic dimension; EDV: end-diastolic volume; ESD: end-systolic dimension; ESV: end-systolic volume; EF: ejection fraction; SV: stroke volume; CO: cardiac output.

Echo parameter	General cohort (n = 181)	CMDS 0 (n = 36)	CMDS 1 (n = 69)	CMDS 2 (n = 57)	CMDS 3 (n = 19)	p_K-W_
		1	2	3	4	
	Me [25-75%]	Me [25-75%]	Me [25-75%]	Me [25-75%]	Me [25-75%]	
LA volume, ml	47.00 [45.00-49.00]	46.00 [45.00-47.00]	47.00 [46.00-49.00]	47.00 [46.00-49.00]	47.00 [46.00-50.00]	0.012
LA dimension, cm	3.50 [3.40-3.60]	3.40 [3.40-3.50]	3.50 [3.40-3.62]	3.50 [3.40-3.70]	3.50 [3.40-3.70]	0.001
End‐diastolic interventricular septal thickness (IVST), cm	0.90 [0.79-0.91]	0.80 [0.78-0.90]	0.90 [0.80-0.96]	0.90 [0.80-1.00]	0.90 [0.80-0.99]	0.016
End‐diastolic LV posterior wall thickness (PWT), cm	0.91 [0.82-1.00]	0.90 [0.81-0.94]	0.96 [0.81-1.00]	0.95 [0.88-1.00]	0.99 [0.90-1.00]	0.028
Relative wall thickness (RWT)	0.40 [0.37-0.43]	0.39 [0.36-0.42]	0.40 [0.37-0.43]	0.40 [0.38-0.42]	0.40 [0.39-0.43]	0.390
LV mass, gm	135.28 [113.83-163.92]	121.63 [102.08-138.44]	135.28 [118.35-162.36]	145.03 [114.82-179.80]	146.76 [116.58-179.80]	0.007
LV mass index (g/m^2^)	72.10 [61.49-83.76]	67.47 [60.91-76.50]	75.22 [63.00-86.85]	77.59 [61.92-87.19]	68.61 [59.07-88.39]	0.194
EDD, cm	4.50 [4.20-4.90]	4.30 [4.10-4.67]	4.60 [4.30-4.90]	4.80 [4.20-4.90]	4.80 [4.20-4.90]	0.084
EDV, ml	78.00 [70.00-79.50]	77.00 [69.00-78.00]	78.00 [69.00-79.00]	78.00 [70.00-87.00]	79.00 [78.00-89.00]	0.004
ESD, cm	2.90 [2.80-3.00]	2.90 [2.73-2.90]	2.90 [2.80-3.00]	2.90 [2.80-3.00]	2.90 [2.80-3.00]	0.273
ESV, ml	29.23 [26.10-31.20]	27.64 [24.93-29.14]	29.41 [25.98-31.20]	30.26 [26.60-32.76]	30.42 [29.23-35.60]	0.002
EF, %	62.00 [61.00-63.00]	63.00 [61.00-64.75]	62.00 [60.00-63.00]	61.20 [60.00-63.00]	62.00 [60.00-62.00]	0.010
SV, ml	47.58 [43.40-50.70]	47.19 [42.37-49.60]	47.40 [43.55-50.70]	48.36 [43.40-52.20]	48.36 [47.40-53.40]	0.090
CO, L/min	3.52 [3.17-4.00]	3.45 [3.09-3.84]	3.51 [3.17-3.95]	3.52 [3.11-4.20]	3.79 [3.38-4.35]	0.062

Spearman’s correlation analysis was performed to study the relationship between cardiometabolic risk factors and echocardiographic parameters. The most significant correlations in terms of strength are shown in Table 5. EDV and ESV were associated with cardiometabolic risk factors and strongly correlated with visceral fat level, waist circumference, and BMI.

**Table 5:** Correlation coefficients of the individual components of cardiometabolic risk factors with key echocardiographic measures Note: rs: Spearman’s correlation coefficient; p: statistical significance. Abbreviations: BMI: body mass index; VAI: visceral adiposity index; HOMA-IR: homeostatic model assessment of insulin resistance; non-HDL-c: - non-high-density lipoprotein cholesterol; SBP: systolic blood pressure; DBP: diastolic blood pressure; CRP: C-reactive protein.

Echo parameter Risk factor		LA volume	EDD	EDV	ESD	ESV	EF	SV
BMI, Kg/m^2^	r_s_	0.461	0.408	0.504	0.266	0.524	-0.358	0.385
	p	0.000	0.000	0.000	0.000	0.000	0.000	0.000
Waist circumference, cm	r_s_	0.432	0.428	0.520	0.290	0.528	-0.338	0.417
	p	0.000	0.000	0.000	0.000	0.000	0.000	0.000
Waist-hip ratio	r_s_	0.286	0.323	0.370	0.214	0.354	-0.191	0.296
	p	0.000	0.000	0.000	0.008	0.000	0.018	0.000
Visceral Fat Level	r_s_	0.471	0.450	0.563	0.343	0.568	-0.351	0.464
	p	0.000	0.000	0.000	0.000	0.000	0.000	0.000
VAI	r_s_	0.234	0.248	0.329	0.029	0.336	-0.229	0.238
	p	0.000	0.002	0.000	0.722	0.000	0.004	0.003
HOMA-IR	r_s_	0.324	0.193	0.365	0.276	0.396	-0.300	0.267
	p	0.000	0.016	0.000	0.001	0.000	0.000	0.001
Insulin, μIU/mL	r_s_	0.340	0.213	0.398	0.247	0.418	-0.291	0.297
	p	0.000	0.008	0.000	0.002	0.000	0.000	0.000
Triglycerides, mmol/L	r_s_	0.232	0.240	0.355	0.060	0.341	-0.169	0.286
	p	0.004	0.003	0.000	0.456	0.000	0.037	0.000
Non-HDL-c, mmol/L	r_s_	0.189	0.285	0.330	0.073	0.343	-0.228	0.237
	p	0.019	0.000	0.000	0.367	0.000	0.005	0.003
Atherogenicity index	r_s_	0.220	0.377	0.367	0.016	0.380	-0.294	0.255
	p	0.006	0.000	0.000	0.840	0.000	0.000	0.001
SBP, mm Hg	r_s_	0.224	0.269	0.412	0.154	0.365	-0.145	0.365
	p	0.005	0.001	0.000	0.056	0.000	0.073	0.000
DBP, mm Hg	r_s_	0.259	0.286	0.366	0.226	0.371	-0.240	0.302
	p	0.001	0.000	0.000	0.005	0.000	0.003	0.000
Mean arterial pressure, mm Hg	r_s_	0.273	0.313	0.416	0.212	0.402	-0.220	0.352
	p	0.001	0.000	0.000	0.008	0.000	0.006	0.000
CRP, mg/L	r_s_	0.165	0.206	0.268	0.089	0.263	-0.165	0.204
	p	0.041	0.010	0.001	0.270	0.001	0.041	0.011

Since the level of visceral fat showed the greatest correlation strength with key echocardiographic parameters as compared to others, we conducted further analysis of echocardiographic parameters based on the presence of this particular risk factor (Table 6) and found that patients with an increased level of visceral fat had higher values for LA volume and size, left ventricular EDD, EDV, and ESV.

**Table 6:** Characteristics of structural and functional parameters of the heart in young adults based on the level of visceral fat Note: n: number of people; Ме: median [interquartile range, 25th–75th percentile], p_1,2_: significance between groups with normal (1) and increased (2) levels of visceral fat calculated using the Mann‐Whitney test. Abbreviations: LA: left atrium; EDD: end-diastolic dimension; EDV: end-diastolic volume; ESV: end-systolic volume; EF: ejection fraction.

Parameter	Group with normal visceral fat level (n = 171)	Group with increased visceral fat level (n = 10)	p_1,2_
	1	2	
	Me [25-75%]	Me [25-75%]	
LA volume, mL	47.00 [45.00-48.00]	49.00 [47.50-51.25]	0.005
LA size, cm	3.50 [3.40-3.60]	3.75[3.58-3.93]	0.000
EDD, cm	4.40 [4.20-4.90]	4.95 [4.80-5.10]	0.001
EDV, mL	78.00 [70.00-79.00]	87.00 [78.00-99.75]	0.007
ESV, mL	29.16 [26.14-31.20]	33.15 [29.72-38.97]	0.010
EF, %	62.00 [61.00-63.00]	61.00 [60.00-62.25]	0.206

Given the significant impact of increased visceral fat levels on left atrio-ventricular sizes and volumes and the fact that the largest proportion of patients with elevated visceral fat levels was found in the CMDS 3 group, we identified a subgroup of individuals with an increased level of visceral fat within the CMDS 3 group. Based on the previous findings, as they had the maximum number of metabolic abnormalities and the highest cardiometabolic risk (CMDS 3), we denoted this new group as CMDS 3-overly high.

**Figure 1:**
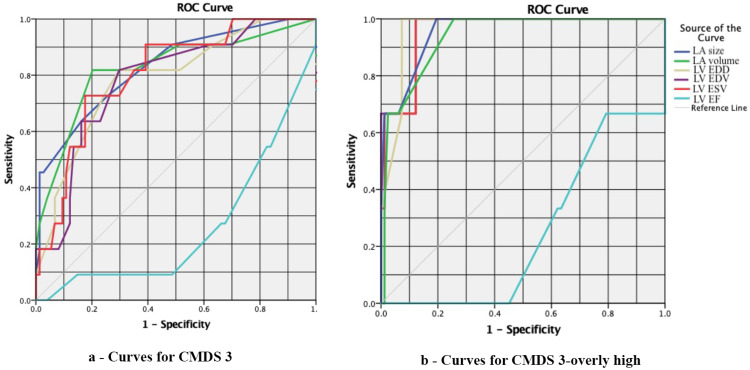
ROC curves for echocardiographic parameters in men with CMDS 3 (panel a) and CMDS 3-overly high (panel b) ROC: Receiver operator characteristic; CMDS: cardiometabolic disease staging.

ROC analysis was performed to determine the cut-off values (Table 7) of echocardiographic parameters associated with overly high cardiometabolic risk (CMDS 3-overly high). Given that the reference values of cardiac structural and functional parameters are sex-dependent, ROC analysis was conducted separately for females and males. When plotting the ROC curves in males, a significant AUC was obtained for the CMDS 3 group, followed by a further increase in the AUC in the CMDS 3-overly high subgroup (Figure 1). Hence, it is fair to say that the increase in hemodynamic changes is directly proportional to the increase in the stages of cardiometabolic risk. The plotting of ROC curves for hemodynamic parameters in women revealed insignificant changes.

**Table 7:** Results of ROC analysis of echocardiography parameters for males in the CMDS 3-overly high subgroup Note: АUC: Area under the curve; CI: 95% confidence interval. Abbreviations: LA: left atrium; EDD: end-diastolic dimension; EDV: end-diastolic volume; ESV: end-systolic volume; ROC: receiver operator characteristic; CMDS: cardiometabolic disease staging.

Parameter	Cut-off	Sensitivity, %	Specificity, %	AUC, %	CI	p
LA volume, mL	49.5	66.7	93.9	93.7	0.843-1.0	0.010
LA size, cm	3.65	100	80.5	95.5	0.877-1.0	0.008
EDD, cm	4.95	100	92.7	96.3	0.914-1.0	0.007
EDV, mL	89.5	100	87.8	95.9	0.888-1.0	0.007
ESV, mL	34.9	100	87.8	95.5	0.885-1.0	0.008

## Discussion

Although several studies have been conducted to discover the effects of cardiometabolic abnormalities on cardiac function, to the best of our knowledge, this is the first effort to use the CMDS system to study structural and functional changes in the heart [20,29-33]. Second, the study population comprised relatively young subjects without cardiometabolic disorders. Although the majority had derangements within the reference limits, these results could be used to identify and set early preventive measures. Finally, based on the results, we identified a new subgroup of patients with CMDS 3 who were at a higher risk (CMDS 3-overly high) of increased cardiac chamber size and volume. 

The CMDS system proposed by Guo et al. is an easy-to-use tool for evaluating the risk of diabetes, cardiovascular disease, and all-cause mortality [27]. Given its simplicity, it can be used in primary care settings to assess and guide interventions for preventing and treating cardiometabolic diseases. Considering that the validation study of the CMDS system included relatively young adults, we decided to use it in our study. 

Our findings, such as the increasing trend in the severity of dysmetabolic processes, various types of obesity, and an increase in BP observed from CMDS 0 to CMDS 3, align with the results of other studies [5,20,31,34,35]. Notably, a substantial increase in LA volume and size, EDV, ESV, IVS thickness, LV posterior wall thickness, and LV myocardial mass occurred with an increase in cardiometabolic risk (Table 3). These results could be explained by an increase in the frequency and severity of obesity, increased BP, prediabetes, and other cardiometabolic changes, as well as their combinations, and, in general, are comparable with those previously reported by us and other researchers [11,36]. 

It should also be noted that among all studied cardiometabolic risk factors, only visceral fat level was associated with all echocardiographic parameters (Table 5) and correlated with other risk factors (r_s_ = 0.238-0.901; p = 0.000-0.003). These data reflect general notions about the relationship between obesity and its types and cardiac structural and functional parameters [34,37-39]. The levels of EDV and ESV correlated with all parameters, reflecting alterations in carbohydrate and lipid metabolism, as well as an increase in BP.

These results allowed us to distinguish and propose a subgroup of CMDS 3-overly high in young adults with CMDS 3. This is further supported by the fact that despite the presence of abdominal obesity in 100% of subjects in CMDS 3, an increased level of visceral fat was found only in a quarter of cases and was associated with an additional increase in dysmetabolic changes (increased levels of triglycerides, C-reactive protein, leptin, insulin, VAI, and HOMA-IR), and increased BP (SBP, DBP, and mean arterial pressure). Excess visceral fat results in the increased synthesis of pro-inflammatory cytokines, such as leptin, and reduces the secretion of protective insulin sensitizing adiponectin [40,41]. This “adiposopathy” results in insulin resistance and hyperinsulinemia, glucotoxicity, and increased angiotensin synthesis, and in conjunction with the increased tone of the sympathetic nervous system, contributes further to myocardial dysfunction [41].

The ROC analysis confirmed a significant increase in the LV ESV and EDV, as well as the left atrial volume in the CMDS 3-overly high group. The ROC curves for females revealed no significant changes. This may be due to the low frequency of elevated visceral fat in the CMDS 3 group (n = 1). 

The main limitation of this study is its cross-sectional design, which makes it impossible to determine causality. Another limitation is generalizability of this study. Although the study was conducted using a validated risk assessment system, since it was conducted in Russia, extrapolation of these results to other regions or countries may not be possible.

## Conclusions

In individuals without established cardiometabolic diseases, the preload and afterload increase simultaneously with an increase in CMDS. Although CMDS is a comprehensive and accurate risk stratification tool, our results suggest that when designing strategies for cardiovascular disease prevention in young adults, apart from CMDS parameters, bioimpedance analysis should be performed to assess the level of visceral fat. This proves helpful in identifying patients who are at a much higher risk (CMDS 3-overly high) of developing heart failure and atrial fibrillation due to atrioventricular remodeling. The results of this study could also be used to identify new dominants or phenotypes of HFpEF. 
